# Serum-Free Suspension Culture of MDCK Cells for Production of Influenza H1N1 Vaccines

**DOI:** 10.1371/journal.pone.0141686

**Published:** 2015-11-05

**Authors:** Ding Huang, Wen-Juan Peng, Qian Ye, Xu-Ping Liu, Liang Zhao, Li Fan, Kang Xia-Hou, Han-Jing Jia, Jian Luo, Lin-Ting Zhou, Bei-Bei Li, Shi-Lei Wang, Wen-Ting Xu, Ze Chen, Wen-Song Tan

**Affiliations:** 1 State Key Laboratory of Bioreactor Engineering, East China University of Science and Technology, Shanghai 200237, China; 2 Shanghai Institute of Biological Products Co., Ltd., Shanghai 200052, China; Penn State University School of Medicine, UNITED STATES

## Abstract

Development of serum-free suspension cell culture processes is very important for influenza vaccine production. Previously, we developed a MDCK suspension cell line in a serum-free medium. In the present study, the growth kinetics of suspension MDCK cells and influenza virus production in the serum-free medium were investigated, in comparison with those of adherent MDCK cells in both serum-containing and serum-free medium. It was found that the serum-free medium supported the stable subculture and growth of both adherent and suspension cells. In batch culture, for both cell lines, the growth kinetics in the serum-free medium was comparable with those in the serum-containing medium and a commercialized serum-free medium. In the serum-free medium, peak viable cell density (VCD), haemagglutinin (HA) and median tissue culture infective dose (TCID_50_) titers of the two cell lines reached 4.51×10^6^ cells/mL, 2.94Log_10_(HAU/50 μL) and 8.49Log_10_(virions/mL), and 5.97×10^6^ cells/mL, 3.88Log_10_(HAU/50 μL), and 10.34Log_10_(virions/mL), respectively. While virus yield of adherent cells in the serum-free medium was similar to that in the serum-containing medium, suspension culture in the serum-free medium showed a higher virus yield than adherent cells in the serum-containing medium and suspension cells in the commercialized serum-free medium. However, the percentage of infectious viruses was lower for suspension culture in the serum-free medium. These results demonstrate the great potential of this suspension MDCK cell line in serum-free medium for influenza vaccine production and further improvements are warranted.

## Introduction

In recent years, animal cell culture technology has gradually replaced the traditional chick embryo production process for influenza vaccine production. Currently, most of cells applied for influenza vaccine production are adherent and grown as monolayers. As a result, large-scale culture processes mainly rely on cultivating adherent cells on microcarriers in serum-containing medium [1–3].

For vaccine production, supplementation of serum brings about many problems, such as high cost, batch variation and risk of contamination with viruses, mycoplasmas and prions [4, 5]. In addition, the presence of serum can cause difficulties for downstream purification [6]. To address these issues, serum-free medium has been exploited in vaccine production processes. Several studies reported successful development of microcarrier-based cell culture processes using serum-free medium for influenza vaccine production [7, 8]. Although a high virus production yield can be obtained through the microcarrier-based approach, it is often challenging for scale-up due to the labor-intensive process and high cost of microcarriers [9, 10]. The use of suspension cells is anticipated to facilitate the scale-up of the production process by eliminating trypsinization and reattachment of cells, which are otherwise required in the microcarrier system [11, 12].

Thus far, several suspension cell lines, including MDCK, PER. C6, AGE. CR, EB14/EB66 and CAP, have been established and applied in influenza vaccine production [10, 12–20]. In particular, for influenza production, serum-free suspension culture of MDCK cells has been reported [9, 18, 19]. However, low influenza virus productivity is generally obtained in simple batch culture, albeit the productivity can be promoted by applying complex fed-batch or perfusion culture [21–23]. Therefore, there is an urgent need to develop more effective batch cell culture process for suspension culture of MDCK cells in influenza vaccine production. Previously, a few studies characterized cell growth and influenza virus production in different culture modes, for example, adherent and suspension cultures in either serum-containing or serum-free medium, in comparison with approaches using chick embryo [12, 24, 25]. However, a direct comparison among different cell culture modes regarding cell growth and influenza virus production is still missing, which should be vital to the development of suspension cell-based influenza vaccine production process.

Previously, we had successfully established a suspension MDCK cell line for influenza virus production process [26]. The objective of the present study was to investigate the growth of MDCK cells during subculture and batch culture in different culture modes, including serum-containing adherent culture, serum-free adherent culture and serum-free suspension culture. The influenza virus production in these batch cultures was also compared.

## Materials and Methods

### Cell lines and culture conditions

The adherent MDCK cells (CCL-34, ATCC) were cultivated on Cytodex^TM^ 3 microcarriers (3 g/L, GE Healthcare) in DMEM (Gibco) supplemented with 10% (v/v) fetal bovine serum (FBS, Gibco) or a proprietary serum-free medium developed by the authors (MDCK-SFM1) [27]. The adherent MDCK cells were adapted to suspension culture by the serum reduction and serial passaging approach in another proprietary serum-free medium developed by the authors (MDCK-SFM2) [26, 27], and the resulting suspension MDCK cells were cultured in MDCK-SFM2 or a commercialized serum-free medium Ex-cell MDCK (Sigma-Aldrich). The formulations of MDCK-SFM1 and MDCK-SFM2 were listed in Tables A and B in S1 File. In addition, both adherent and suspension MDCK cell lines were cryopreserved in State Key Laboratory of Bioreactor Engineering (East China University of Science and Technology Shanghai, China) and can be available upon request for research purpose.

Adherent and suspension cells were subcultured every 2 days in 150-cm^2^ T-flasks (Corning) and 125-mL shaker flasks (Corning), respectively. For subculture, cell densities of adherent and suspension cells were maintained at 4×10^4^ cells/cm^2^ and 3×10^5^ cells/mL, respectively. The incubator was controlled at 37°C with 5% CO_2_. For batch culture and infection experiments, adherent or suspension cells were seeded at 3×10^5^ cells/mL in either serum-containing or serum-free medium as above in stirred bioreactors (3 L, Applikon). The starting culture volume was 1 L and agitation was 80 rpm. Dissolved oxygen was controlled at 50% saturation. Culture temperature was set at 37°C and pH was controlled at 7.0 during cell growth phase, while both were shifted to 35°C and 7.2 during virus production phase, respectively.

### Cell counting

Determination of viable cell density (VCD) on microcarriers was performed every 12 h using the crystal violet method as described before [28]. VCD in suspension culture was determined by trypan blue dye exclusion assay [29]. Specific growth rate (μ) was calculated based on Eq (1).

$$\mu =({\text{Ln VCD}}_{2}\text{-}{\text{Ln VCD}}_{1})/({\text{t}}_{2}\text{-}{\text{t}}_{1})$$ (1)

Wherein, VCD_1_ and VCD_2_ were the viable cell density at the time point of t_1_ and t_2_, respectively. Specific death rate (Sdr) equals to the absolute value of μ during virus production phase.

### Infection of MDCK cells

At the time of infection (TOI), for serum-containing culture, medium was discarded and cells were rinsed twice with phosphate buffered saline (PBS). Cells on the microcarriers were resuspended in DMEM containing TPCK-trypsin (5 mg/L, Sigma-Aldrich), and then infected with influenza virus A/ California/ 7/ 2009 (H1N1) (NIBSC) with multiplicity of infection (MOI) of 0.01. For serum-free culture, TPCK-trypsin and influenza virus were delivered to bioreactors directly at TOI without PBS rinse.

### Determination of virus titers

Total titer of influenza virus was measured by HA assay [30]. Specially, fresh chicken red blood cells were obtained from the Shanghai Institute of Biological Products Co., Ltd, and set at 2×10^7^ cells/mL. HA Titers were expressed as Log_10_HA units per test volume (Log_10_HAU/50μL). Assuming one chicken red blood cell being sufficient for the agglutination of one virus particle, HA titers could be converted into concentration of total viruses (CTV, virions/mL) using the concentration of chicken red blood cells added for HA titers by Eq (2).

$$\text{CTV}=2\times 10^{7}\times 10^{\left(\text{HA titer}\right)}$$ (2)

Concentration of infectious viruses (CIV, virions/mL) were determined by median tissue culture infective dose (TCID50) assay [31]. The cell-specific virus yield (Svy) was calculated as described by Bock et al. [7].

### Statistical analysis

All values were presented as mean ± standard deviation (n = 3). Statistical analyses were performed with a two-tailed Student’s t-test, and differences between samples were considered as statistically significant when p<0.05.

## Results

### Comparably stable passaging of MDCK cells under different culture conditions

**Fig 1:**
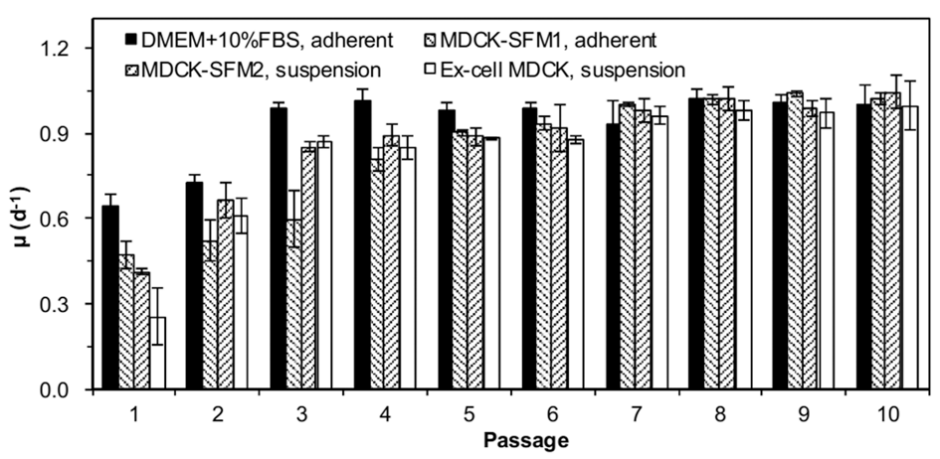
MDCK cell growth stability in different cultures. Adherent MDCK cells were seeded at 4×10^4^ cells/cm^2^ in 150 cm^2^ T-flasks, and suspension cells were seeded at 3×10^5^ cells/mL in 125 mL shaker flasks. Cells were subcultured every 2 days for 10 passages after thawing and average specific growth rates were monitored.

To evaluate the adaptability of MDCK cells, cell growth was investigated in serum-containing adherent, serum-free adherent and serum-free suspension culture modes for 10 passages. Serum-free medium for suspension culture included Ex-cell MDCK and MDCK-SFM2 medium. The specific growth rate (μ) was calculated and summarized in Fig 1. In all cultures, the specific growth rates gradually increased with passaging and all reached a stable level within 10 passages. However, it took 5 passages for both suspension and adherent MDCK cells in serum-free medium to achieve the stable growth, which was 2 more passages than that for adherent cells in serum-containing medium (3 passages). At the steady stage, no difference in the growth rates was found in different culture modes. Hence, the suspension MDCK cell lines in the proprietary serum-free medium could be continuously passed with a comparable stability to other culture modes.

### Better growth of suspension MDCK cells in MDCK-SFM2 in batch culture without infection

**Fig 2:**
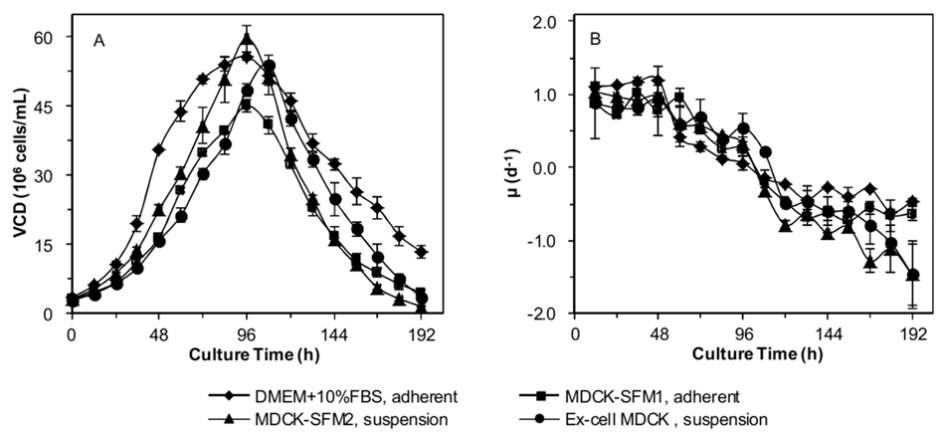
Time courses of (A) viable cell density (VCD) and (B) specific cell growth rate (μ) of MDCK cells in different batch cultures. MDCK cells were seeded at 3×10^5^ cells/mL in 3 L stirred bioreactors.

Batch culture of MDCK cells was also performed in the same four different modes, including serum-containing adherent culture in DMEM plus 10% FBS, serum-free adherent culture in MDCK-SFM1, serum-free suspension cultures in MDCK-SFM2 and Ex-cell MDCK. VCD and μ were determined every 12 h and their time profiles were shown in Fig 2. In general, shortly after inoculation, cells in all cultures began to propagate steadily, and no lag phase was present. Before 72 h, adherent and suspension cells in serum-free medium grew slower than adherent cells in serum-containing medium. Afterwards, growth of adherent cells in serum-containing adherent culture slowed down and μ turned negative. Cells in serum-free adherent culture, suspension culture in MDCK-SFM2 and serum-containing adherent culture reached peak VCD at 96 h and thereafter, VCD began to decrease and μ dropped below zero. However, for serum-free suspension culture in Ex-cell MDCK medium, peak VCD appeared at 120 h and then μ became negative. As listed in Table 1, there was no significant difference in peak VCD among adherent cells in serum-containing medium, suspension cells in MDCK-SFM2 and suspension cells in Ex-cell MDCK (p>0.05), while the peak VCD of adherent cells in serum-free medium was less than others (p<0.05). By comparing adherent cells in serum-containing medium and MDCK-SFM1, no significant difference in the average of μ before the appearance of peak VCD, expansion fold and doubling time was detected, respectively (p>0.05). In addition, suspension cells in MDCK-SFM2 and Ex-cell MDCK and adherent cells in serum-containing medium had similar values of all parameters (p>0.05). Notably, there was significant difference for these values when comparing suspension cells in MDCK-SFM2 with adherent cells in MDCK-SFM1 and the peak VCD, expansion fold, and the average μ before the appearance of peak VCD of the former were 32.31%, 27.14%, 8.94% higher, respectively, and doubling time of the former was 21.36% shorter (p<0.05). In summary, the suspension MDCK cells grew slightly better in MDCK-SFM2 than in other three modes.

**Table 1:** MDCK cell growth kinetics in batch culture in different culture modes.

	Peak VCD (10^6^ cells/mL)	Time of peak VCD (h)	Avg. μ before time of peak VCD (d^-1^)	Expansion fold (fold)	Double time (h)
A	5.58±0.09	96	0.69±0.03	15.82±1.61	12.20±1.24
B	4.51±0.12	96	0.67±0.01	14.69±0.51	13.08±0.46
C	5.97±0.28	96	0.73±0.01	18.68±0.62	10.28±0.29
D	5.40±0.19	108	0.66±0.04	19.27±3.16	11.36±1.87
P value A-B	0.010	n.a.	0.444	0.167	0.445
P value A-C	0.206	n.a.	0.139	0.648	0.152
P value A-D	0.346	n.a.	0.302	0.648	0.398
P value B-C	0.022	n.a.	0.016	0.018	0.017
P value B-D	0.030	n.a.	0.181	0.333	0.615
P value C-D	0.142	n.a.	0.165	0.505	0.103

A, DMEM with 10% FBS, adherent; B, MDCK-SFM1, adherent; C, MDCK-SFM2, suspension; D, Ex-cell MDCK, suspension.

### Similar growth characteristics of MDCK cells in different batch cultures post infection

**Fig 3:**
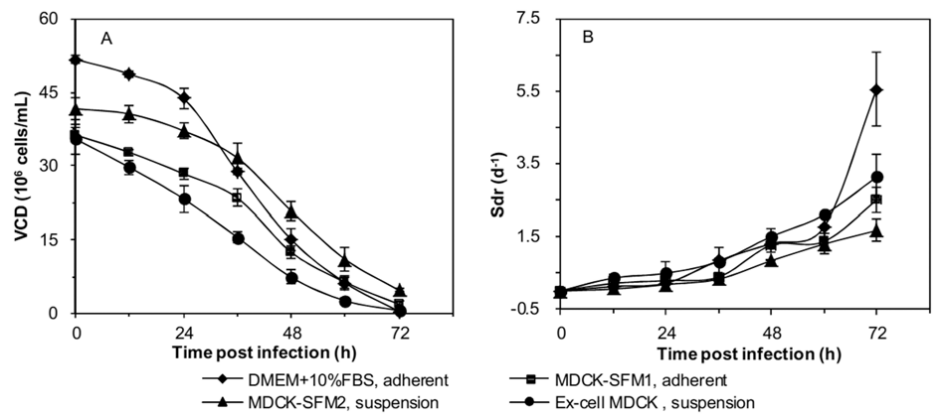
Time courses of (A) viable cell density (VCD) and (B) specific cell death rate (Sdr) of MDCK cells in different batch cultures post infection. MDCK cells were seeded at 3×10^5^ cells/mL in 3 L stirred bioreactors, and infected with influenza virus after cultured for 72 h.

The growth of MDCK cells in batch culture post infection was also investigated among the same four culture modes, and VCD and average specific death rate (Sdr) were compared, respectively (Fig 3A). At 0 h_p.i_, VCD of adherent cells in serum-containing medium and MDCK-SFM1 and suspension cells in MDCK-SFM2 and Ex-cell MDCK were 51.70×10^5^, 36.29×10^5^, 41.63×10^5^ and 35.44×10^5^ cells/mL, respectively. For adherent cells in serum-containing medium, VCD declined slowly within 0–24 h_p.i_ (Sdr: 0.17 d^-1^). And itdropped much faster during 24–72 h_p.i_ (Sdr: 2.37 d^-1^). For adherent cells in MDCK-SFM1 and suspension cells in MDCK-SFM2, the average Sdr were 0.29 and 0.18 d^-1^ within 0–36 h_p.i_, and 1.71 and 1.26 d^-1^ within 36–72 h_p.i_, respectively. And, no apparent turning point was noticed for suspension cells in Ex-cell MDCK, with an overall average Sdr of 1.40 d^-1^ within 0–72 h_p.i_. Therefore, the death of MDCK cells in serum-free medium was lower than serum-containing medium post infection, with suspension cells in MDDK-SFM2 the lowest.

### Better production of influenza virus in batch culture for suspension culture in MDCK-SFM2

**Fig 4:**
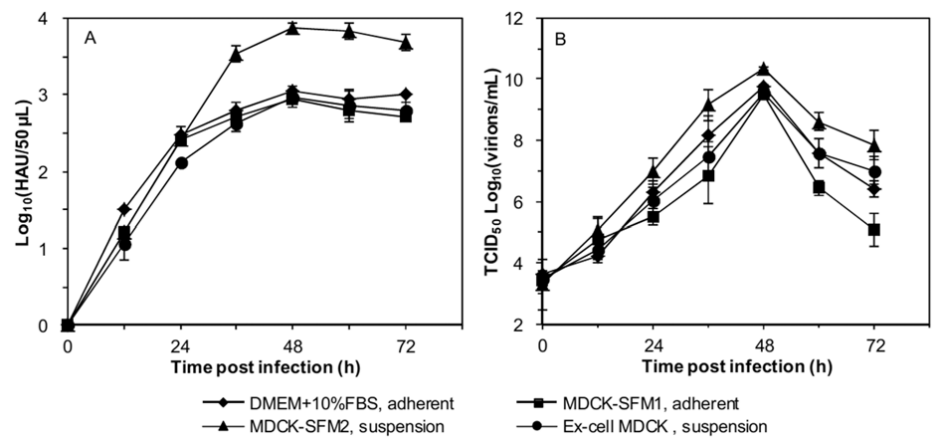
Time courses of (A) HA titers and (B) TCID_50_ titers of influenza virus in different batch cultures post infection.

Production of influenza virus in batch culture within 72 h was measured. In all culture modes, the time profiles of HA titers were similar showing a quick increase and reaching the plateau at 36 h (Fig 4A). However, the maximum HA titers varied and were 3.05Log_10_ (HAU/50μL), 2.94Log_10_ (HAU/50μL), 3.88Log_10_ (HAU/50μL) and 2.97Log_10_ (HAU/50μL for serum-containing adherent culture, serum-free adherent culture, suspension culture in MDCK-SFM2 and suspension culture in Ex-cell MDCK, respectively. Accordingly, the maximum HA titer of suspension culture in MDCK-SFM2 was 27.21%, 31.97 and 30.64% higher than those of serum-containing adherent culture, serum-free adherent culture, and suspension culture in Ex-cell MDCK, respectively (p<0.05). The time profiles of TCID_50_ titers also followed a similar trend, with peak TCID_50_ titers of 9.76Log_10_(virions/mL), 8.49Log_10_(virions/mL), 10.34Log_10_(virions/mL) and 9.53Log_10_(virions/mL), respectively (Fig 4B). The peak TCID_50_ titer of suspension culture in MDCK-SFM2 was 6.00%, 9.01 and 5.50% higher than those of other three cultures, respectively (p<0.05). Additionally, as summarized in Table 2, the concentration of total viruses (CTV) in suspension culture in MDCK-SFM2 was 6.74, 8.65, and 8.00 folds, the concentration of infectious viruses (CIV) was 3.85, 7.04 and 6.44 folds, and Svy was 8.42, 7.61 and 6.78 folds of those in serum-containing adherent culture, serum-free adherent culture, and suspension culture in Ex-cell MDCK, respectively (p<0.05). However, the ratio of CIV to CTV (R_I/T_) in serum-containing adherent culture was the highest, and that in suspension culture in MDCK-SFM2 the lowest. Collectively, the productivity of influenza virus of suspension MDCK culture in MDCK-SFM2 was better than of other three culture modes, despite a low R_I/T_.

**Table 2:** Influenza virus production in batch culture in different culture modes.

	CTV (10^10^ virions/mL)	CIV (10^9^ virions/mL)	Svy (10^3^ virions/cell)	R_I/T_ (%)
A	3.18±0.34	5.72±0.84	6.14±0.57	17.97±0.70
B	2.47±0.56	3.13±0.95	6.79±1.28	12.53±0.99
C	21.40±2.93	22.02±3.57	51.70±9.90	10.27±0.26
D	2.68±0.37	3.42±0.66	7.61±1.70	12.74±0.74
P value A-B	0.271	0.124	0.577	0.024
P value A-C	0.013	0.013	0.023	0.024
P value A-D	0.293	0.095	0.362	0.018
P value B-C	0.012	0.015	0.024	0.089
P value B-D	0.714	0.728	0.636	0.836
P value C-D	0.012	0.009	0.025	0.047

A, DMEM with 10% FBS, adherent; B, MDCK-SFM1, adherent; C, MDCK-SFM2, suspension; D, Ex-cell MDCK, suspension. CTV, concentration of total viruses; CIV, concentration of infectious viruses; Svy, cell-specific virus yield; R_I/T_, ratio of CIV to CTV.

## Discussion

Based on our previous studies [26, 27], the four different culture modes for applied in the present study (serum-containing adherent culture in DMEM plus 10% FBS, serum-free adherent culture in MDCK-SFM1, and serum-free suspension cultures in MDCK-SFM2 and Ex-cell MDCK) were studied and compared. The growth of MDCK cells and production of influenza virus were investigated in these cultures, including adherent serum-containing culture, adherent serum-free culture and suspension cultures in two different serum-free medium. It was demonstrated that our own proprietary serum-free medium (MDCK-SFM1 and MDCK-SFM2) supported the stable subculture and growth of adherent and suspension MDCK cells, although 2 more passages were needed to reach a stable maximum specific growth rate (μ) compared to adherent cells in serum-containing medium. In batch culture, the growth kinetics of MDCK cells in MDCK-SFM1 (adherent) and MDCK-SFM2 (suspension) was similar to that in adherent serum-containing culture and suspension culture with the commercialized serum-free medium Ex-cell MDCK. Notably, suspension culture in MDCK-SFM2 supported quicker cell proliferation and a higher peak VCD and slowed down cell death, suggesting better cell growth. Moreover, the HA and TCID_50_ titers, CTV, CIV and Svy of suspension culture in MDCK-SFM2 were higher than in other three cultures, indicating the efficient production of influenza virus.

The influenza H1N1 virus strain used in the present study was an influenza A/PR8/34 virus backbone containing virus. In literature, the peak HA titer and peak TCID_50_ of influenza A/PR8/34 virus in MDCK cells were reported to be 3.31Log_10_(HAU/50 μL) [32] and 9.37Log_10_(virions/mL) [9] in serum-containing adherent batch culture, 3.08Log_10_(HAU/50 μL) and 9.38Log_10_(virions/mL) in serum-free adherent perfusion culture [23], and 3.54Log_10_(HAU/50 μL) and 10.11Log_10_(virions/mL) in serum-free suspension perfusion culture [18], respectively. For other cell lines, including PER. C6 cells [14], AGE1.CR cells [33], HEK-293 cells [34] and human CAP cells [20], 1.80~3.40log_10_(HAU/50 μL) and 7.89~10.72Log_10_(virions/mL) were achieved in serum-free suspension perfusion culture. In the present study, for suspension culture of MDCK cells in MDCK-SFM2, 3.88Log_10_(HAU/50 μL) and 10.34Log_10_(virions/mL) were obtained for the peak HA titer and peak TCID_50_, respectively, suggesting that the culture process developed was very efficient in virus production.

Several potentials factors can be attributed to the improved virus production for MDCK cells in the proprietary serum-free medium (MDCK-SFM2). In literature, gene expression of *ST6GAL1* has been considered as an indicator of influenza virus propagation in MDCK cells [35, 36]. In fact, several studies have successfully improved the influenza virus yield by upregulating glycan receptors on cell surface [35, 37]. In the present study, it was found that the expression level of suspension MDCK cells in MDCK-SFM2 was higher than that of adherent cells in serum-containing culture, which might potentially contribute to the observed high virus yield (Figure A in S1 File.). However, the expression level for cells in MDCK-SFM2 was lower than that in Ex-cell MDCK (Figure A and Table C in S1 File) which implicates that other factors may also be important for virus production in these culture modes, such as the medium composition. Nevertheless, the medium (MDCK-SFM2) used for suspension MDCK cell culture was quite different from MDCK-SFM1 and Ex-cell MDCK, which may contribute to the observed higher virus yield in MDCK-SFM2 than in MDCK-SFM1 and Ex-cell MDCK. For influenza vaccine production, nutritional demand of cells would be dramatically changed, so the nutrients such as glucose and relevant amino acids should be very important [38]. As a result, the maintaining medium used in virus production processes might not be able to provide sufficient nutrients to meet the requirements for achieving a high virus productivity [9, 23, 32].

Analysis of the ratio of infectious to total virus concentration (R_I/T_) showed a 5.44% reduction when comparing serum-containing culture to serum-free culture, and a further 2.26% reduction from adherent to suspension culture. This has also been observed by others [9]. In the present study, the infectious progeny viruses released from suspension culture of MDCK cells in MDCK-SFM2 was faster than in other cultures (see Fig 4B), and the resulting more progeny virus probably resulted in a relatively higher MOI for the proceeding infection and replication process. As previously reported, a higher MOI and corresponding defective interfering particles (DIPs) could lead to a lower R_I/T_ [39, 40]. Further, non-infectious cell-killing particles (niCKPs) caused by apoptosis, necrosis and cell lysis that evoked by multifarious causes (e. g. serum absence, fast infection and replication of influenza virus, nutritional imbalance resulted by high cell density and high viral yield) in serum-free medium might also contribute to this phenomenon [39, 41].

In summary, a MDCK suspension cell culture was evaluated for production of influenza H1N1 A virus. The growth of the suspension MDCK cell line in the serum-free medium MDCK-SFM2 was found similar to that in the adherent serum-containing culture as well as suspension culture with a commercialized serum-free medium. Production of the influenza H1N1 virus was significantly improved with suspension culture in MDCK-SFM2. Albeit a lower ratio of infectious viruses to total virus concentration was observed, this issue is expected to be addressed through further optimization of medium components and operating strategies. Hence, the serum-free suspension culture process is promising for efficient production of influenza vaccine.

## Supporting Information

S1 File Figure A. Gene expression of *ST6GAL1* in different batch cultures. Table A. Formulation of SFM1. Table B. Formulation of SFM2. Table C. Primers and reaction condition for real-time PCR
(DOCX)
