# Supplementary material for: Serum-Free Suspension Culture of MDCK Cells for Production of Influenza H1N1 Vaccines
Source: PLoS One. 2015 Nov 5;10(11):e0141686. doi: 10.1371/journal.pone.0141686 (PMC4634975; doi:10.1371/journal.pone.0141686)
Supplement: S1 File — Table A. Formulation of SFM1. Table B. Formulation of SFM2. Table C. Primers and reaction condition for real-time PCR (DOCX) [file pone.0141686.s001.docx]

**Supporting Information**

**Materials and methods**

**Determination of *ST6GAL1* gene expression by quantitative real time-PCR**

RNA was extracted by Trizol Reagent (Invitrogen life technologies), and cDNA was obtained by reverse transcription using Super Script^TM^ III Reverse Transcriptase (Invitrogen). Real-time PCR was performed with 2×PCR master mix (Array Star) on ViiA 7 Real-time PCR System (Applied Biosystems) with conditions as follows: 10 min at 95^o^C, 40 circles of 10 s at 95^o^C, and 60 s at 60^o^C. The primer sequences and reaction conditions for Real-time PCR were provided in **S3 Table**.

**Results**

***ST6GAL1* gene expression**

The expression level of *ST6GAL1* in MDCK cells under the four culture modes were determined and shown as *ST6GAL1*/*β-actin* (**S1 Fig**.). The expression levels of MDCK cells in serum-free cultures with MDCK-SFM1, MDCK-SFM2 and Ex-cell MDCK was higher than that in serum-containing culture. However, the expression level in MDCK-SFM2 was lower than in Ex-cell MDCK.

**Fig A.** Gene expression of *ST6GAL1* in different batch cultures

**Table A** Formulation of SFM1

| **Component** | **Concentration** (mg/L) |
| --- | --- |
| Cupric Sulfate Pentahydrate | 15.97 |
| Ferric Nitrate Nonahydrate | 20.02 |
| Ferrous Sulfate Heptahydrate | 0.417 |
| Magnesium Chloride Hexahydrate | 61.2 |
| Magnesium Sulfate | 48.84 |
| Calcium Chloride Dihydrate | 154.5 |
| Potassium Chloride | 311.8 |
| Sodium Bicarbonate | 1200 |
| Sodium Chloride | 6996 |
| Dibasic Sodium Phosphate | 71.02 |
| Sodium Dihydrogen Phosphate | 54.3 |
| L-Alanine | 4.45 |
| L-Arginine Hydrochloride | 147.5 |
| L- Asparagine Monohydrate | 7.5 |
| L-Aspartic cid | 6.65 |
| L-Cystine Dihydrochloride | 17.56 |
| L- Cysteine Hydrochloride Monohydrate | 31.29 |
| L-Glutamic Acid | 7.35 |
| L-Glutamine | 292 |
| Glycine | 18.75 |
| L-Histidine Hydrochloride Monohydrate | 31.48 |
| L-Isoleucine | 54.47 |
| L-Leucine | 59.05 |
| L- Lydine hydrochloride | 91.25 |
| L-Methionine | 17.24 |
| L-Phenylalanine | 35.48 |
| L-Proline | 17.25 |
| L-Serine | 26.25 |
| L-Threonine | 53.45 |
| L-Tryptophan | 9.02 |
| L-Tyrosine Disodium Dihydrate | 55.79 |
| L-Valine | 52.85 |
| D-Biotin | 0.0035 |
| Folic Acid | 2.66 |
| Niacinamide | 2.02 |
| Pyridoxine Hydrochloride | 2 |
| Thiamine Hydrochloride | 2.17 |
| Lipoic Acid | 0.105 |
| D-Glucose | 3151 |
| Sodium Pyruvate | 55 |
| Hypoxanthine | 2.1 |
| Thymidine | 0.365 |
| Hydroxyethyl Piperazine Ethanesulfonic Acid | 3574.5 |
| Cholesterol | 3.87 |
| DL-alpha-Tocopherol Acetate | 0.03 |
| Myristic Acid | 0.005 |
| Palmitic Acid | 0.005 |
| Palmitoleic Acid | 0.005 |
| Stearic Acid | 0.005 |
| Tween 80 | 1.1 |
| Transferrin | 10 |
| Bovine Serum Albumin | 100 |
| Insulin | 10 |
| Sodium Selenite | 81.47 |
| Lucaratone Tryptone USP | 2100 |
| Sodium Phenol Red | 8.63 |

**Table B** Formulation of SFM2

| **Component** | **Concentration** (mg/L**)** |
| --- | --- |
| Cupric Sulfate Pentahydrate | 15.97 |
| Ferric Nitrate Nonahydrate | 40.04 |
| Ferrous Sulfate Heptahydrate | 0.6 |
| Magnesium Chloride Hexahydrate | 61.2 |
| Magnesium Sulfate | 48.84 |
| Calcium Chloride Dihydrate | 154.5 |
| Potassium Chloride | 311.8 |
| Sodium Bicarbonate | 1200 |
| Sodium Chloride | 6996 |
| Dibasic Sodium Phosphate | 71.02 |
| Sodium Dihydrogen Phosphate | 54.3 |
| L-Alanine | 4.45 |
| L-Arginine Hydrochloride | 147.5 |
| L- Asparagine Monohydrate | 7.5 |
| L-Aspartic cid | 6.65 |
| L-Cystine Dihydrochloride | 17.56 |
| L- Cysteine Hydrochloride Monohydrate | 31.29 |
| L-Glutamic Acid | 7.35 |
| L-Glutamine | 584 |
| Glycine | 18.75 |
| L-Histidine Hydrochloride Monohydrate | 31.48 |
| L-Isoleucine | 54.47 |
| L-Leucine | 59.05 |
| L- Lydine hydrochloride | 91.25 |
| L-Methionine | 17.24 |
| L-Phenylalanine | 35.48 |
| L-Proline | 17.25 |
| L-Serine | 26.25 |
| L-Threonine | 53.45 |
| L-Tryptophan | 9.02 |
| L-Tyrosine Disodium Dihydrate | 55.79 |
| L-Valine | 52.85 |
| D-Biotin | 0. 0035 |
| Folic Acid | 2.66 |
| Niacinamide | 2.02 |
| Pyridoxine Hydrochloride | 2 |
| Thiamine Hydrochloride | 2.17 |
| Lipoic Acid | 0. 105 |
| D-Glucose | 4500 |
| Sodium Pyruvate | 110 |
| Hypoxanthine | 2.1 |
| Thymidine | 0.365 |
| Hydroxyethyl Piperazine Ethanesulfonic Acid | 3574.5 |
| Cholesterol | 3.87 |
| DL-alpha-Tocopherol Acetate | 0.07 |
| Myristic Acid | 0.01 |
| Palmitic Acid | 0.01 |
| Palmitoleic Acid | 0.01 |
| Stearic Acid | 0.01 |
| Tween 80 | 2.2 |
| Transferrin | 10 |
| Bovine Serum Albumin | 100 |
| Insulin | 10 |
| Sodium Selenite | 81.47 |
| Lucaratone Tryptone USP | 2100 |
| Pluronic F68 | 1000 |
| Sodium Phenol Red | 8.63 |

**Table C** Primers and reaction condition for real-time PCR

| **Gene** | **Sequence of primers** | **Annealing temperature**  **(^o^C)** | **Amplicon size (bp)** |
| --- | --- | --- | --- |
| *β-actin* | \| F:5’AAGTCCTTTGCCTTCCCAA3’ \| \| --- \| \| R:5’TCTCTTTCCCTCCCCTGTG3’ \| | 60 | 89 |
| *ST6GAL1* | \| F:5’ATGGGACCCGTCTGTTTACC3’ \| \| --- \| \| R:5’GCACACATCAGTCTTACGCTTAG3’ \| | 60 | 292 |
